# The impact of margin reduction on radiation dose distribution of ultra‐hypofractionated prostate radiotherapy utilizing a 1.5‐T MR‐Linac

**DOI:** 10.1002/acm2.14179

**Published:** 2023-11-27

**Authors:** Cem Onal, Esma Efe, Recep Bozca, Cagdas Yavas, Guler Yavas, Gungor Arslan

**Affiliations:** ^1^ Faculty of Medicine Department of Radiation Oncology Baskent University Ankara Turkey; ^2^ Adana Dr. Turgut Noyan Research and Treatment Center Department of Radiation Oncology Baskent University Faculty of Medicine Adana Turkey

**Keywords:** dosimetry, MR‐linac, prostate cancer, radiotherapy, stereotactic body radiotherapy

## Abstract

**Background:**

We examined the effects of reducing the planning target volume (PTV) margin in MR‐guided radiotherapy (MRgRT) on the distribution of radiation dose to target volumes and organs‐at‐risk (OARs). Thus, we compared MR‐Linac (MRL) plans with and without reduced margin and intensity‐modulated radiotherapy (IMRT) plan with conventional linac for low‐risk prostate cancer patients receiving 36.25 Gy in five fractions of ultra‐hypofractionated radiation therapy.

**Materials and Methods:**

Twenty low‐risk prostate cancer patients treated with 1.5 T MR‐Linac were evaluated. The same planning CT images were used for four plans: the MRL‐R plan with reduced margin planning target volume (PTV‐R) and the MRL‐N plan with normal margin PTV (PTV‐N), which is also used for IMRT plan. In four plans, PTV doses, organs‐at‐risk (OARs) doses, the homogeneity index (HI), and monitor units were compared.

**Results:**

All plans met the criteria for PTV coverage and OARs dose constraints. The maximum and mean PTV doses were significantly higher in the MRL‐R and MRL‐N plans compared to the IMRT plan. The HI was lowest in the IMRT plan (0.040 ± 0.013) and highest in the MRL‐N plan (0.055 ± 0.012; *p* < 0.001). There was no significant difference in the PTV dosimetric parameters between the MRL‐R and the MRL‐N plans. The high doses in the rectum was significantly lower in the MRL‐R compared to other plans. The bladder V36.25 Gy was significantly lower in the MRL‐R plan (2.43 ± 1.87 Gy) compared to MRL‐N (4.50 ± 2.42 Gy; *p* < 0.001), and IMRT plans (4.76 ± 2.77 Gy; *p* < 0.001). There was no significant difference in the low‐dose volumes of the body, maximum femur doses, or monitor units across each plan.

**Conclusions:**

Ultra‐hypofractionated MR‐guided RT with 1.5 T MRL is dosimetrically feasible for patients with prostate cancer. The improved soft tissue contrast and the online adaptive plan for 1.5 T MR‐Linac allows for PTV margin reduction resulted in a significant dose reduction in OARs.

## 1 INTRODUCTION

Several innovations have led to improved radiotherapy (RT) techniques over the past decade, including intensity‐modulated RT (IMRT), volumetric modulated arc therapy (VMAT), and image‐guided RT. The evolution of stereotactic body radiation therapy (SBRT) over the past decade has allowed for utilizing these innovations to test ultra‐hypofractionated radiation therapy schedules with only five fractions. Based on long‐term changes in bowel and urinary system, the RTOG 0938 study confirms that the 5 ‐(7.25 Gy per fraction in 2 weeks) or 12‐fractions (4.3 Gy per fraction in 2.5 weeks) regimens are well tolerated.^1^, ^2^

The clinical introduction of magnetic resonance (MR)‐guided linear accelerators (MRL) has significantly impacted RT workflows by enabling MR‐imaging prior to and during beam‐on. Furthermore, by performing interfraction plan adaptation, these systems can counteract anatomical changes between treatment fractions, such as rotation and deformations of the targets and organs‐at‐risk (OARs).^3^ As a result of real‐time imaging during treatment and of an enhanced soft tissue contrast, it has been suggested in recent MR‐guided RT (MRgRT) studies to reduce the planning target volume (PTV) margin for the prostate by up to 3 mm.^4^

Compared to treatment planning for a conventional linac, treatment planning for an MRL is more difficult due to the design of the machine. During treatment planning, in addition to the presence of a magnetic field, several MRL‐specific beam and collimator characteristics must be considered. At the isocenter plane, the Multi‐Leaf Collimator (MLC) in the MR‐Linac system has a leaf width of 7.15 mm, which is greater than the 5 mm leaf width typically found in conventional linear accelerators. In addition, the Unity system (Elekta AB, Stockholm, Sweden) does not support collimator rotation. To meet all MRL‐specific requirements without sacrificing plan quality, it is necessary to develop and to validate treatment planning techniques. In previous studies, MRL and conventional treatment planning for prostate cancer patients was found to have a comparable quality.^5^, ^6^, ^7^, ^8^

In this study, we examined the potential impact of reducing the PTV margin in the context of MRgRT on radiation dose distribution for both the target volumes and OARs. Hence, our objective was to conduct a comparative analysis between MRL plans with and without reduced margin, and conventional linac IMRT plans for patients with low‐risk prostate cancer, utilizing the ultra‐hypofractionated radiation therapy regimen of 36.25 Gy administered over five fractions.

## 2 MATERIALS AND METHODS

### 2.1 Patient selection

Twenty patients with low‐risk prostate cancer treated previously with ultra‐hypofractionated RT with 1.5 T MRL between January 2021 and November 2021 were evaluated retrospectively. All patients provided their written permission to use their data after they were de‐identified for educational and scientific purposes.

### 2.2 Target volumes

The patients underwent a computed tomography (CT) scan to determine dose distribution and a diagnostic MRI, which included diffusion‐weighted imaging (DWI), dynamic contrast‐enhanced (DCE), and high spatial resolution T2‐weighted (T2W) images. The patients were instructed to have an empty bowel and a full bladder during simulation and treatment.^9^ The diagnostic MRI and the planning CT images were registered using a deformable registration technique.

**FIGURE 1:**
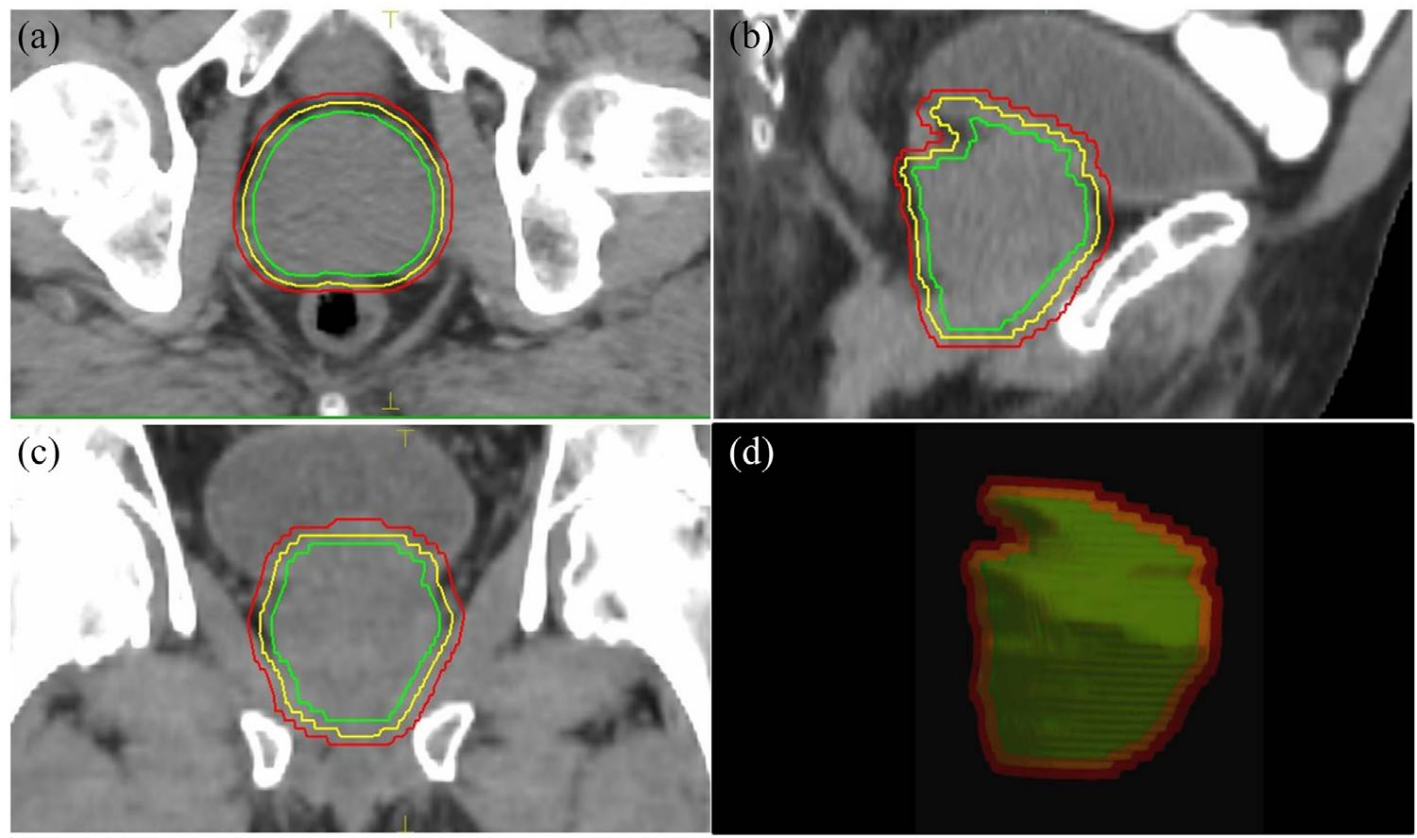
Prostate (green line), PTV with reduced margin (yellow line) and PTV with standard margins demonstrated in (a) axial, (b) sagittal, (c) coronal CT slices, and (d) beams‐eye‐view reconstruction.

The CTV including only the prostate, and the normal PTV (PTV‐N) was created by expanding the CTV by 6 mm in all directions, except posteriorly, where a 5‐mm margin was used, and the PTV with reduced margin (PTV‐R) was defined as a 3‐mm expansion in all directions of the CTV for the MRL treatment plans (MRL‐R) (Figure 1). The OARs included the rectum, bladder, femoral heads, and urethra. The rectum was delineated as extending from the anal verge to the rectosigmoid junction.^10^

### 2.3 Treatment planning

The prescribed dose was 36.25 Gy delivered in five fractions. Two plans were computed using the Unity MRL‐specific Monaco treatment planning system version 5.40.01, taking into account the 1.5‐T magnetic field using a GPU‐based Monte Carlo dose calculation platform (GPUMCD).^11^ The MRL‐R plan was generated using PTV‐R, which is utilized in clinical practice during treatment with ultra‐hypofractionated MRgRT, and the MRL‐N plan was generated using PTV‐N, which is also used for IMRT plan. All MRgRT plans were created using the “step‐and‐shoot” technique, which is the only IMRT technique currently available in the Unity MRL system. On the original CT dataset, 12 co‐planar field IMRT plans were generated for each treatment plan. All plans were computed utilizing the 1.5‐T‐MRL (Unity® MR Linac System, Elekta AB, Stockholm, Sweden) with 7 MV flattening filter‐free (FFF) photons, 0.2 cm grid spacing, and 2% statistical uncertainty per control point.

The IMRT plans included 12 co‐planar fields with the same gantry angles used in MRL plans. The IMRT plan was created using the Monaco treatment planning system version 5.51.10 with the Monte Carlo algorithm and a dose planner post‐optimization for delivery on Elekta Versa HD Linac with 6 MV energy.

Both systems use the same dose‐volume goals and priorities to ensure that the PTV has adequate doses, while OARs receiving safe doses. The plan was optimized to ensure that at least 95% of the prescribed dose was administered to PTV. Less than 1% of the volume received more than 107% (V107) of the prescribed dose. Table 1 provides a summary of the dose constraints for OARs. The target volumes receiving 95% (V95) and 107% (V107) of the prescribed dose were calculated. The target homogeneity index (HI) was calculated as:

$$HI\;=\left[\left(D2-D98)/D50\right)\right]$$

where *D*2 and *D*98—the minimal doses of 2% and 98% of the target volumes, respectively—were used as surrogates for the maximum and minimum doses.^12^ A greater HI value indicated a poorer homogeneity of the dose distribution.

**TABLE 1:** Dose constraints for organs at risk.

Organs	Constraints
PTV	V36.25 Gy ≥ 95%
Rectum	V36.25 Gy < 2% V36 Gy < 5% V32.63 Gy < 10% V29 Gy < 20% V27.19 Gy < 25% V18.13 Gy < 50%
Bladder	V36.25 Gy < 5% V18.13 Gy < 40%
Femur	D1cc < 20 Gy
Urethra	D5% < 38 Gy V (42 Gy) ≤ 0.03 cc

The doses administered to the PTV, OARs, and HI were compared between the treatment plans. The D*n* and V*n* were calculated for PTV and OARs. V*n* represents the percentage of organ volume receiving ≥ *n*Gy, and D*n* is the percentage of the organ receiving *n*% of the prescribed dose. V36.25 Gy, V32.63 Gy, V27.19 Gy, and V18.13 Gy as well as D1cc and mean doses were compared between plans for the rectum and bladder. V36Gy, V29Gy, and V27.19 Gy were additional rectum dose volume parameters calculated in the four plans. D1cc was used for the maximum femur doses. Total body volumes V20Gy and V10Gy were chosen to evaluate the low dose exposure.

### 2.4 Statistical analysis

For the statistical analysis, SPSS 22.0 (SPSS for Windows, IBM Corp., Armonk, NY, USA) and MedCalc version 20.111 (MedCalc Software Ltd., Ostend, Belgium) were used. SPSS was used for statistical analysis and MedCalc for image generation. The one‐way analysis of variance (ANOVA) and Wilcoxon signed‐rank test were used to determine the significance of differences in PTV and OARs doses between the plans. The Friedman test was used to compare more than two treatment plans. The Friedman test was used to compare more than two treatment plans. Because a maximum of three different subgroups were compared, significance was accepted in pairwise comparisons at *p* < 0.02. All reported *p* values are two‐sided, with *p* < 0.05 considered statistically significant.

## 3 RESULTS

### 3.1 Patients

The median age and serum PSA levels were 70 years (range 55−74 years) and 6.9 ng/mL (range 1.1−9.7 ng/mL), respectively. Four patients (20%) had clinical T1c disease, 12 (60%) had T2a disease, and four (20%) had T2b disease. The median prostate volume was 51.2 cm^3^ (range 15.9–87.5 cm^3^). The median PTV‐N and PTV‐R were 113.6 (44.5−163.6 cm^3^) and 79.2 cm^3^ (range 28.8−123.8 cm^3^), respectively.

### 3.2 Target volume doses

**FIGURE 2:**
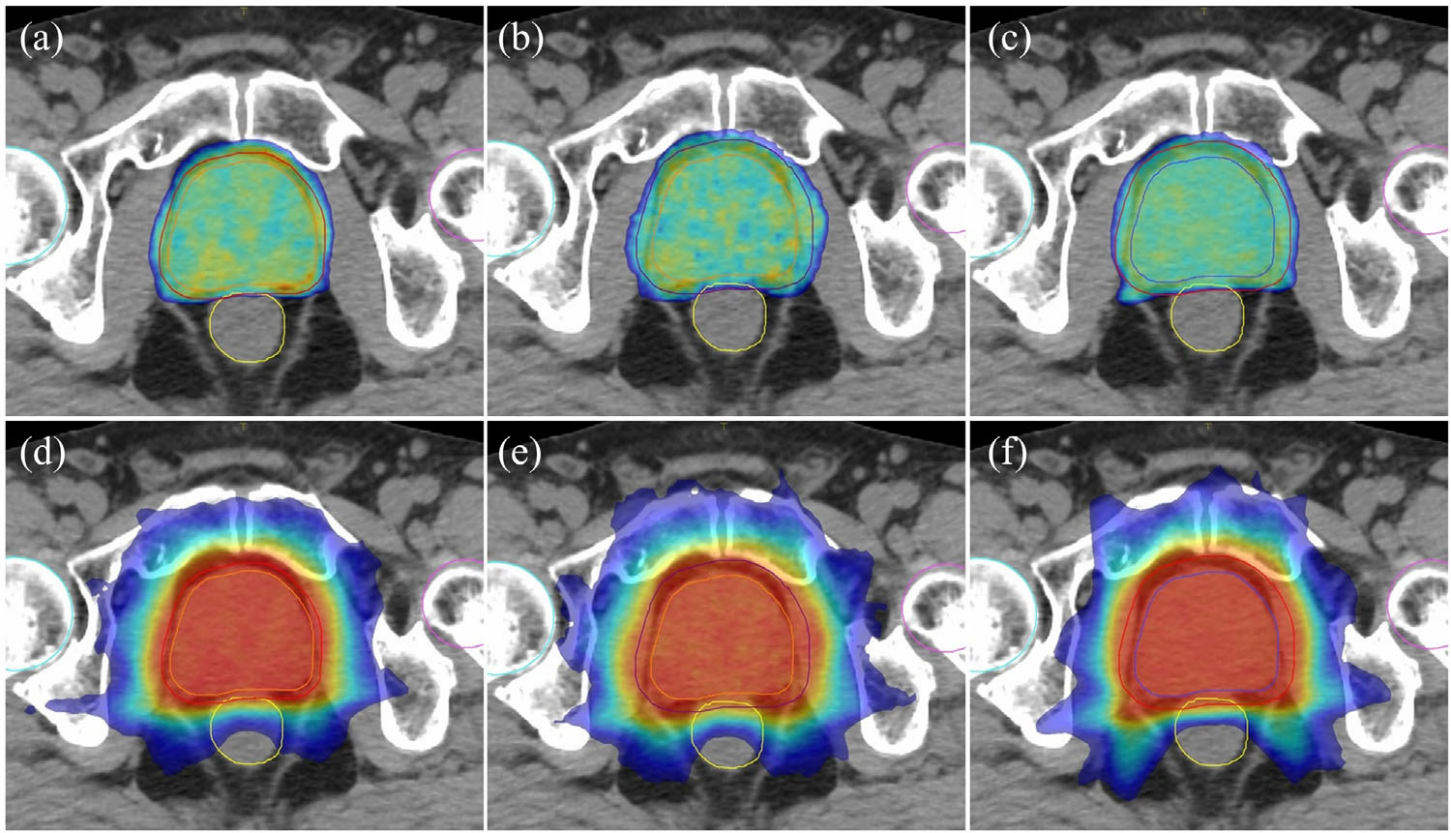
Representative axial CT slices showing 95% of prescribed dose distributions (green area) for the (a) MR‐Linac plan with reduced margin (MRL‐R), (b) MR‐Linac plan with normal margin (MRL‐N), and (c) intensity‐modulated radiotherapy plan and 50% of prescribed dose distributions (blue area) for the (d–f) same consecutive plans (red area represents 95% isodose area).

All plans met the criteria for PTV coverage (Table 2), and Figure 2 shows the planning CT axial sections depicting the PTV dose distributions for the MRL‐R, MRL‐N, and IMRT plans.

**TABLE 2:** Planning target volume doses according to four different plans.

	MRL‐R	MRL‐N	IMRT
PTV			
V107 (%)	0.006 ± 0.014	0.066 ± 0.224	0.009 ± 0.004
V95 (%)	99.99 ± 0.42	99.93 ± 0.10	99.87 ± 0.16
Dmean (Gy)	37.11 ± 0.20	37.07 ± 0.19	36.76 ± 0.19
D2% (Gy)	37.91 ± 0.27	37.94 ± 0.34	37.31 ± 0.35
D98% (Gy)	36.13 ± 0.27	35.90 ± 0.22	35.83 ± 0.19
HI	0.048 ± 0.011	0.055 ± 0.012	0.040 ± 0.013
MU	1959 ± 134	2127 ± 193	2389 ± 322
Body			
V20 Gy (%)	0.78 ± 0.32	1.12 ± 0.46	0.89 ± 0.37
V10 Gy (%)	5.24 ± 2.05	6.68 ± 2.54	6.30 ± 2.35

Abbreviation: Dn, percent of organ receiving n% of the prescribed dose; HI, homogeneity index; IMRT, intensity modulated radiotherapy; MRL‐N, MR‐linac plan with normal margins; MRL‐R, MR‐linac plan with reduced margins; MU, monitor unit; PTV, planning target volume; Vn, percentage organ volume receiving ≥ nGy.

There was no significant difference on average V107 and V95 values for PTV between the MRL‐N, and IMRT plans in the first scenario, for which plans were generated using PTV‐N; however, the mean dose and D2 of PTV in MRL‐N were significantly higher than in the IMRT plan (*p* < 0.001). There was no statistically significant difference in the D98 value between the plans. The HI was lowest in the IMRT plan (0.040 ± 0.013) which means better dose homogeneity and highest in the MRL‐N plan representing worse dose homogeneity (0.055 ± 0.012; *p* < 0.001).

The V95 value for the MRL‐R plan was significantly higher than the value calculated for the IMRT plan (*p* = 0.02), but there was no significant difference between the other plans. The mean and maximum PTV doses were significantly higher in the MRL‐R plan than in the IMRT plan (*p* < 0.001), whereas the minimum PTV doses differed significantly between the MRL‐R and IMRT plans (*p* = 0.001). There was no significant difference between the MRL‐N and MRL‐R plans in terms of PTV doses and HI.

### 3.3 Organs at risk doses

**FIGURE 3:**
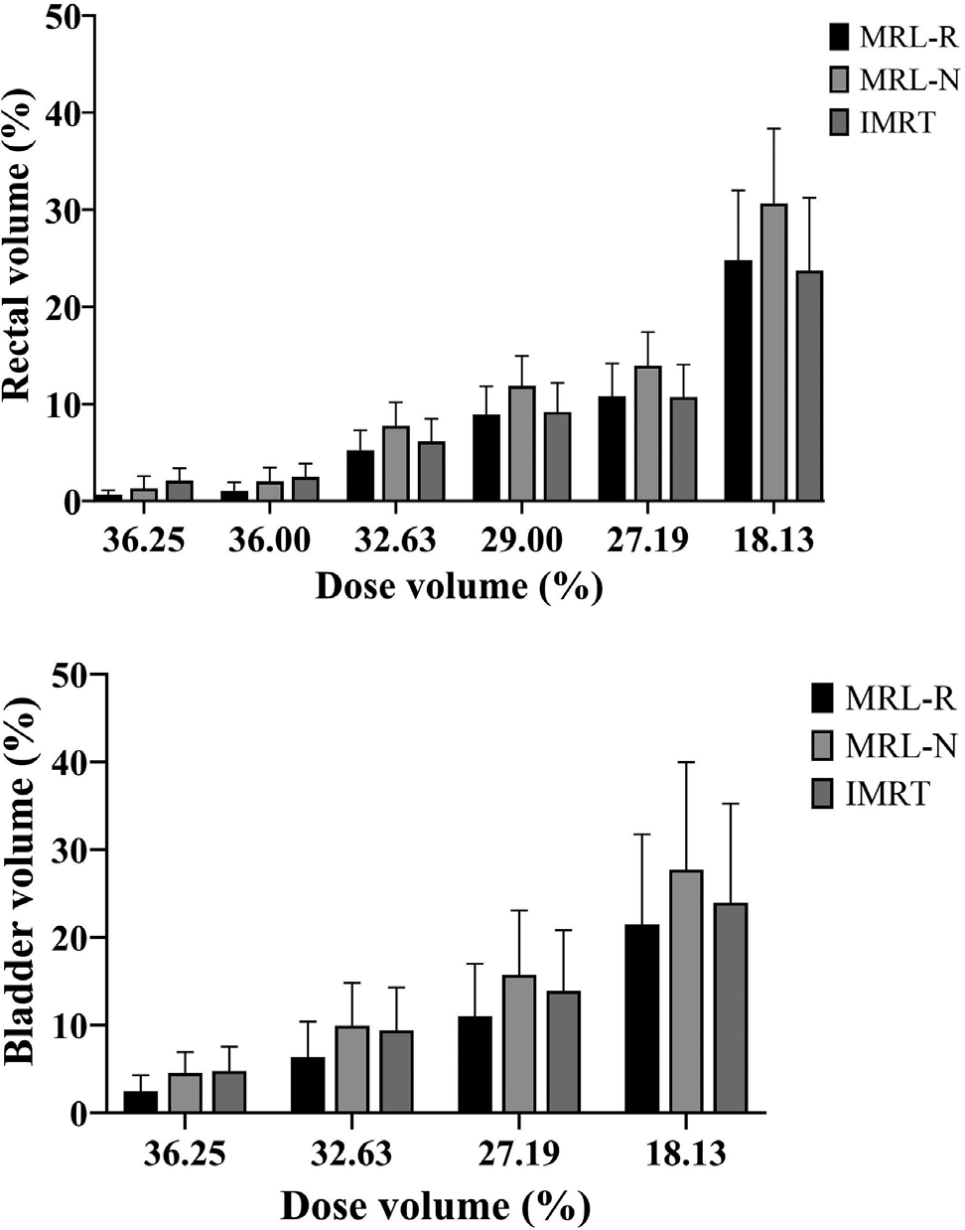
Box and whisker plot demonstrating rectum and bladder doses according to the dose volume parameters across each plan.

**FIGURE 4:**
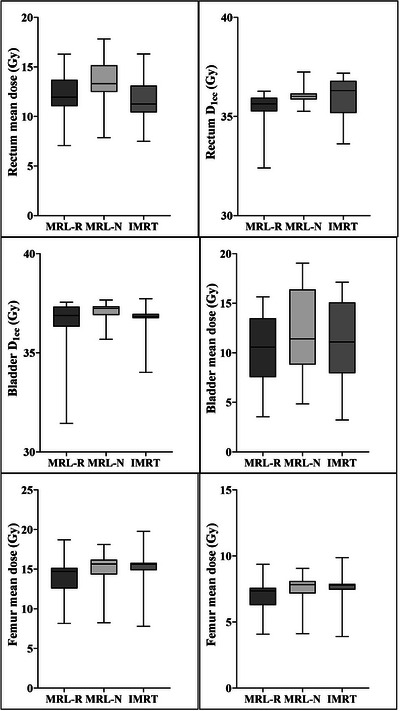
Box and whisker plot demonstrating rectum, bladder and femur doses received by 1 cc volume and mean doses measured in four different plans.

All plans complied with the dose constraints for the rectum, bladder, and femurs. The V36.25 Gy and V36 Gy for the rectum were 0.63 ± 0.49 Gy and 1.06 ± 0.87 Gy, respectively, in the MRL‐R plan, which were significantly lower than those calculated for the MRL‐N (1.29 ± 1.28 Gy and 2.01 ± 1.44 Gy; *p* = 0.001) and IMRT (2.09 ± 1.28 Gy and 2.49 ± 1.38 Gy; *p* < 0.001) plans (Figure 3). Other dose volume parameters for the rectum did not differ significantly across plans. Similarly, no significant difference in D1cc and the mean rectum doses was found between the plans (Figure 4).

The only statistically significant difference was in bladder V36.25 Gy, which was significantly lower in the MRL‐R plan (2.43 ± 1.87 Gy) than was calculated for the MRL‐N (4.50 ± 2.42 Gy; *p* < 0.001), and IMRT (4.76 ± 2.77 Gy; *p* < 0.001) plans (Figure 3). Other dose volume parameters in the bladder as well as the D1cc and mean bladder doses did not differ significantly between plans (Figure 4).

The mean urethra D5 for MRL‐R, MRL‐N and IMRT plans were 37.1 ± 0.9 Gy, 37.0 ± 0.8 Gy, and 36.9 ± 0.8 Gy, respectively, with no statistically significant difference between the plans. The mean femur doses for the MRL‐R, MRL‐N, and IMRT plans were 7.08 ± 1.06 Gy, 7.58 ± 1.06 Gy, and 7.55 ± 1.08 Gy, respectively, with no statistically significant difference between the plans. There was no statistically significant difference in the body's low‐dose volumes (V10 Gy and V20 Gy) received across plans. Although the MRL‐R plan had fewer MUs than the MRL‐N, and IMRT plans, there was no statistically significant difference between the plans (Table 2).

## 4 DISCUSSION

In this treatment planning study, we demonstrated that 1.5‐T MRL is feasible in delivering ultra‐hypofractionated RT with a fraction dose of ≥5 Gy to prostate cancer patients with acceptable target and OARs doses. Due to the capacity of the margin reduction with the online plan adaptation during treatment, the MRL plan provided lower doses to the rectum and bladder which is particularly important in preventing potential complications associated with RT. Due to differences in machine design, such as collimator and MLC configurations, couch movement, and beam energy, the MRL plan exhibited a slight decrease in quality compared to conventional linacs.^13^ Furthermore, the TPS observer experience and the presence of a magnetic field had little effect on plan quality, as evidenced by the negligible differences between MRL plans and conventional linac plans. Thus, ultra‐hypofractionated RT with a 1.5 T MRL for prostate cancer is a technically and dosimetrically feasible method.

Ultra‐hypofractionated RT with a total dose of 36.25 Gy delivered in five fractions to the entire prostate gland was previously associated with acceptable levels of acute and late toxicity.^1^, ^14^ Better image quality than with CT, the online adaptive therapy, and real‐time cross‐sectional imaging are just some of benefits that MRgRT offers due to its recent integration into linear accelerators.^15^ Because MRL can adjust the dose distribution daily and can perform imaging with a high soft tissue contrast during beam delivery, recent studies have suggested and have used a 3‐mm PTV margin for the prostate.^4^ A recent study has suggested the use of prostate cancer treatment with MRgRT in a single‐center retrospective series^16^, ^17^; however, the geometry of an MRL differs from that of conventional linacs, which may have an effect on dose distribution and may limit the available treatment techniques.

Previous studies have demonstrated the viability of MRgRT for treating a variety of cancers,^18^, ^19^, ^20^ including prostate cancer.^5^, ^6^, ^7^, ^8^ The majority of these studies demonstrated that reduced margins and plan adaptation result in an equivalent dose to the target and a reduced dose to OARs; however, the results of the studies vary due to the use of different MRL systems, treatment planning systems, dose and fractionations, and RT techniques (Table 3). Park et al.^8^ compared conventional linac‐based VMAT plans to the MRIdian ^60^Co system (MR‐^60^Co), where smaller PTV margins for the MR‐^60^Co ‐based IMRT technique were used, and found that the quality of MR‐^60^Co ‐based IMRT plans is comparable to the quality presented by VMAT plans. Furthermore, for MR‐^60^Co plans, lower doses to the rectum and bladder were observed. This finding aligns with our previous study, where we identified reduced margin as the cause for the lower rectum and bladder high doses in the MRL plan. Van de Schoot et al.^7^ evaluated the feasibility of the 1.5‐T MRL treatment plan compared to the current VMAT plan in a moderate hypofractionation scheme with a total dose of 64.6 Gy delivered in 19 fractions. Despite minor differences in plan quality between the MRL and VMAT plans due to differences in machine characteristics, their findings support the clinical implementation of MRL treatment planning for prostate cancer patients. In the current study, the maximum and mean PTV doses were found to be higher in 1.5‐T MRL plans compared to the conventional IMRT plan and no significant difference in OARs doses when normal PTV margins were utilized; however, the high doses in the rectum and bladder were significantly reduced in the MRL plan compared to other plans when smaller PTV margins were used. These results support the clinical advantage of online adaptive planning and the high soft tissue contrast of 1.5‐T MRL versus the conventional linac. Moreover, MRL plans appear to be superior to other plans for sparing surrounding organs due to the reduced margin.

**TABLE 3:** Published studies comparing the MR‐linac plan and conventional linacs.

Author (year)	N of patients	RT dose	TPS	Findings
Park (2107)	20	30.6/50.4 Gy	Eclipse Viewray MRIdian	No difference in target volume doses. Maximum rectum and bladder doses were higher in VMAT plan than MRL plan
Christiansen et al. (2018)	20	56/78 Gy/39 fx	Pinnacle 14 Monaco 5.19.03	No difference in target volume doses. With the MRL plan, bladder doses were reduced while bowel and penile bulb doses were increased
Van de Schoot (2019)	8	64.6 Gy/19 fx	Pinnacle 9.10 Monaco 5.4	Increased mean, median and D1% of PTV with MRL plan Worse plan homogeneity with MRL plan
Da Silva Mendes (2021)	20	74–76 Gy/37−38 fx	Viewray MRIdian Monaco 5.11.01	Higher minimum and maximum PTV doses in MRL plan. MRL plan with reduced margin provides better surrounding organ doses. Dose homogeneity similar, dose conformity is marginally better in VMAT plan
Current study	20	36.25 Gy/5 fx	Monaco 5.40.01 Monaco 5.51.10	Higher minimum and mean PTV doses in MRL plan. MRL plan with reduced margin provides better surrounding organ doses

This study has some limitations. The scope of our research includes treatment planning using MRL, and IMRT plans. However, clinical decisions must involve the consideration of the efficacy and toxicity of ultra‐hypofractionated MRgRT with a longer follow‐up. It is also crucial to note that such studies carry the risk of bias, particularly when clinical plans created with a well‐established technique are compared to plans created with a novel technique. Moreover, we evaluated the MRL‐N, and IMRT plans with standard PTV margins, but we generated the MRL‐R plan only with a reduced PTV margin. Despite these limitations, our study is significant because it demonstrates the dosimetric feasibility of MRL with a reduced margin in similar treatment planning systems, and the plans were created by the same experienced physicists who have more than 10 years of experience with the Monaco treatment planning system. The results of this study were consistent, and all the MRL plans were deemed clinically acceptable and comparable to the clinical plans.

## 5 CONCLUSION

This study has demonstrated that it is possible to produce clinically acceptable ultra‐hypofractionated SBRT plans for patients with low‐risk prostate cancer in the presence of a 1.5‐T magnetic field on the MRL. A PTV margin reduction causes significant dose reduction in the rectum and bladder, especially at high‐dose levels, due to an improved soft tissue contrast and an online adaptive plan in MRL. This significant difference may give prostate cancer patients the option of increasing their radiation dose. Although this study supports the dosimetric feasibility of 1.5‐T MRgRT, more research on different planning systems is needed as well as long‐term follow‐ups to determine the clinical effects of ultra‐hypofractionated MRgRT in prostate cancer patients.

## AUTHOR CONTRIBUTIONS

Cem Onal: Design of the work, analysis, drafting, final approval, Esma Efe: Interpretation of data for the work, final approval, Recep Bozca: Interpretation of data for the work, final approval, Cagdas Yavas: Drafting, final approval, Guler Yavas: Drafting, interpretation of data for the work, final approval, Gungor Arslan: Interpretation of data for the work, final approval.

## CONFLICT OF INTEREST STATEMENT

The authors declare that there are no conflicts of interest.

## ETHICS STATEMENT

All procedures performed in studies involving human participants were in accordance with the ethical standards of the institutional and/or national research committee and with the 1964 Helsinki Declaration and its later amendments or comparable ethical standards. Informed consent was obtained from all individuals included in the study.

## Data Availability

Data available on request from the corresponding author.
